# Nutritional knowledge, nutritional status and associated factors among pregnant adolescents in the West Arsi Zone, central Ethiopia

**DOI:** 10.1038/s41598-024-57428-w

**Published:** 2024-03-22

**Authors:** Adane Tesfaye, Yohannes Adissu, Dessalegn Tamiru, Tefera Belachew

**Affiliations:** 1 Department of Nutrition, School of Public Health, College of Medicine and Health Science, Dilla University, Dilla, Ethiopia; 2 Department of Nutrition and Dietetics, Faculty of Public Health, Institute of Health, Jimma University, Jimma, Ethiopia

**Keywords:** Nutritional knowledge, Nutritional status, Pregnant adolescent, West Arsi Zone, Health care, Medical research

## Abstract

When pregnancy occur among teenagers; there is a competition for nutrients between the still-growing adolescent mother and her fetus. Pregnant adolescents’ nutrition issues are not addressed well and changes are too slow in Ethiopia. This study aimed to study, nutrition knowledge, nutritional status and associated factors among pregnant adolescents in West Arsi , central Ethiopia. We conducted a cross-sectional study of 426 pregnant adolescents between January 1 and January 25, 2023. Data were collected using kobo collect and analyzed using SPSS version 25. We performed linear regression to identify independent predictors of nutritional status and multivariable logistic regression analyses to identify nutritional knowledge. Odds ratios (ORs) with 95% confidence intervals were estimated to show the strength of the association. Magnitude of good nutrition knowledge was 23.7%, 95% CI (21.4–25.3%), and the odds of having good nutrition knowledge was 7.5 times higher among participants whose education level was above college compared with illiterate participants [(AOR = 7.5, 95% CI = (5.27–9.38)],the odds of having good nutrition knowledge was 8 times higher among adolescent who had ANC visits, [(AOR = 8, 95% CI = (3.63–13.85)], and the odds of having good nutrition knowledge was 5 times higher among adolescents who received nutrition education [(AOR = 5, 95% CI = (3.67- 13.53)]. Receiving nutrition education (β = 0.25, *P* = 0.002) and good nutrition knowledge (β = 0.08, *P* < 0.001) were positively associated with nutritional status; however, food insecurity (β = − 0.93, *P* < 0.001) was negatively associated with nutritional status. The nutrition knowledge of pregnant adolescents was suboptimal; educational status, ANC visits and nutrition education were associated with good nutrition knowledge, whereas food insecurity, low nutrition knowledge, and not receiving nutrition education were predictors of poor nutritional status. Nutritional education interventions, increasing utilization of ANC, and interventions for improving food security are strongly recommended.

## Introduction

Nutrition is key for unlocking the potential of investment in the overall health of women, children and teenagers. It is fundamental to protect females’ health and nutritional status at every stage of life to guarantee the nutrition and wellbeing of their offspring^1,2^. One of the most important and changeable factors affecting the health of the adolescent mother carrying a fetus, as well as the chronic conditions that affect adults, is thought to be her nutritional status. Teenagers' long-term health trajectory may be altered by dietary behaviors that are positively influenced by good nutritional knowledge^3,4^. Ensuring a robust biological basis for the present and future health, welfare, and productivity of women is contingent upon receiving adequate nutrition during the critical phase of adolescent growth and development^5^.

The World Health Organization (WHO) defines adolescence as the period between the ages of 10 and 19 years. Adolescents who are pregnant are more likely to experience a number of unfavorable growth and developmental outcomes, which can have an adverse effect on mothers’ risk of early cessation of linear growth^6^, and her offspring's risk of low birth weight and small for gestational age^7^. According to research conducted in low- and middle-income countries (LMICs), teenage girls may even cease growing as a result of becoming pregnant^6,7^.

In females, physiological conditions such as pregnancy and lactation increase the likelihood of nutritional risks. Childbearing during adolescence has been found to hinder the postmenarcheal linear and ponderal growth of young girls during the potential window of opportunity for catch-up growth in an undernourished population^8–10^. When the pregnant diet does not supply the required nutrients for her needs or for those of the fetus, the fetal requirements are met by withdrawing these nutrients from the tissues of the pregnant mother. This tissue depletion weakens the mother and increases the probability of serious complications^9,11–14^.

Nutritional knowledge is essential for ensuring that pregnant individuals receive proper nutrition and maintain good health^15^. A lack of knowledge can lead to malnutrition and poor health^16^. One of the components of adopting a healthier nutritional practice is having knowledge about nutrition and health, which is indicative of dietary habit modification. Pregnant adolescents are therefore expected to possess the knowledge essential to maintain optimum health and nutrition during pregnancy^17^.

Several studies have been conducted in Ethiopia in relation to pregnant adolescent nutrition. For example, a study conducted in West Arsi revealed a very high magnitude of undernutrition (34%) among pregnant adolescents^18^, and a study in southern Ethiopia showed a 26.4% magnitude of undernutrition^19^. Moreover, a study in Arsi on pregnant adults showed that participants have limited knowledge and poor dietary diversity practices^20^. There are efforts in nutrition intervention programs and strategies in Ethiopia^21,22^; however, pregnant adolescents’ nutritional issues have not been well addressed, and changes have been made too slow.

A cross sectional study in the Ledzokuku-Krowor Municipality, Ghana showed that, less than half (44.9%) of the pregnant adolescents have high nutritional knowledge and about 19.4% of them have good eating habits^23^, similar study in Mandera Kenya showed that less than half of the mothers (47.5%) had moderate nutrition knowledge and Only 13.5% had a high knowledge^24^.

Most related studies have focused on pregnant mothers in general, with little focus on those who are adolescents, and most of these studies have been conducted in developed countries, with only a few in developing countries^25,26^, where a large percentage of the problem occurs. Insufficient attention has been given to the nutritional knowledge and nutritional status of pregnant adolescents. Nutritional knowledge affects dietary practices and nutritional status. Investigating nutritional knowledge and nutritional status among pregnant adolescents is important when designing appropriate interventions; therefore, this study aimed to evaluate nutritional knowledge, nutritional status and associated factors among pregnant adolescents in the West Arsi Zone, central Ethiopia.

## Methods and materials

### Study setting and design

We conducted a community-based cross-sectional study among pregnant adolescents in West Arsi, central Ethiopia. The zone is in the Oromia region, 250 km from Addis Ababa, Ethiopia’s capital city. The West Arsi Zone has 16 districts. The total population was estimated to be 2,929,894 in mid-2022. The zone covers an area of 12,556 sq.km, with a climate of 45.5% highland, 39.6% midland, and 14.9% lowland. Agriculture is the dominant means of livelihood in this zone^27^. According to the Zonal report of 2022, there are 417 public health facilities, 5 of which are hospitals, 324 of which are health posts, 88 of which are health centers, and 203 of which are private medium and higher clinics, including one nongovernmental hospital and two private hospitals providing health services. At least one sexual and reproductive health (SRH) service was utilized in the zone in 2019, for which 58.6% of the population was utilized [102, 103]. This study was conducted between January 1 and January 25, 2023.

### Source population and study population

All pregnant adolescents in the West Arsi Zone were the source population, whereas those in the selected kebeles were the study population.

### Inclusion criteria

The inclusion criterion for pregnant adolescents was gestational age prior to 16 weeks of pregnancy, as this information is the baseline of a cluster-randomized controlled community trial that was registered with Pan African Clinical Trials.gov under the identification code PACTR202203696996305 and with the title "Effect of nutritional behavioral change communication intervention on maternal outcomes among pregnant adolescents in the West Arsi Zone, Central Ethiopia: Cluster Randomized Controlled Trial".

### Exclusion criteria

Pregnant adolescents who were diseased or unable to provide data at the time of data collection were excluded.

### Sample size determination and sampling technique

The sample size was determined using a single-population proportion formula. with the assumption of a 95% confidence level (Z α/2) and a 0.05 expected margin of error (d). proportion (p) of good nutritional knowledge among pregnant women in Ambo Town, Ethiopia (33%)^28^.

$$\begin{gathered} {\text{n}} = \frac{{({\text{Z}}\alpha /{2})^{{2}} .{\text{ P }}\left( {{1} - {\text{P}}} \right)}}{{\left( {\text{d}} \right)^{{2}} }} = \frac{{({1}.{96})^{{2}} . \, 0.{33}\left( {{1} - 0.{33}} \right)}}{{(0.05)^{2} }} \hfill \\ {\text{n}} = {3}.{84} \times \, \left( {0.{33} \times 0.{67}} \right)/0.00{25 } \approx {339} \hfill \\ \end{gathered}$$

This is part of an RCT study reporting baseline findings; the sample size for other objectives was 459 [PACTR202203696996305]. ], which was greater than 339; therefore, the final sample size was 459.

Pregnant teenagers were identified using a multistage clustered sampling method. In the first stage, four districts were randomly selected from the zone's sixteen districts. In the second phase, 16 kebeles (clusters), the lowest administrative unit, were chosen randomly from the districts that were considered. Subsequently, house-to-house visits were made to each of the selected kebeles to count pregnant adolescents to identify pregnant adolescents who qualified for particular clusters by asking about their most recent menstruation and confirmation via a pregnancy test. This study was conducted on all eligible pregnant adolescents. All pregnant adolescents in the selected kebeles were included in the study, with an average cluster size of 29.

### Data collection procedure and measurements

To obtain this information, we employed a pretested structured questionnaire. The questionnaire asked about health service use, frequency of meals, sociodemographic characteristics, household decision-making, the Household Food Insecurity Access Scale (HFIAS), and nutritional knowledge. Six clinical nurses and two master of public health (MPH) holders worked as supervisors and data collectors, respectively. The pregnancy tests were conducted by three female laboratory technicians. To complete the questionnaire, the data collectors conducted in-person interviews with participants at their homes. To the greatest extent possible, other family members were not permitted to enter the site of the interviews to preserve the adolescent's privacy.

The MUAC (mid-upper arm circumference (MUAC)) was measured using inelastic insertion-type tape from each adolescent mother’s left arm at the midpoint between the tips of the shoulder (olecranon process) and elbow (acromion process). The MUAC was measured while the adolescent stood up relaxed with her hand hanging down. After checking that the tape was applied with the correct tension (neither too loose nor too tight), the MUAC of the adolescent was read and recorded to the nearest 0.1 cm. MUAC measurements were taken with a pair of data collectors following the recommended measurement technique^29^.

Dietary intake was calculated using the 24-h recall method according to the United States Agency for International Development (USAID) and the Food and Nutrition Technical Assistance (FANTA) III recommendations of the Food and Agricultural Organization (FAO) to assess whether the diets of pregnant teenagers were adequately diverse^30^. According to the recommendations, there are ten food groups, including grains, meat, nuts and seeds; eggs; plantains; white roots; tubers; pulses (peas, beans, and lentils); dairy, poultry and fish; dark green leafy vegetables; and other fruits and vegetables rich in vitamin A, other vegetables and other fruits. A pregnant adolescent was considered to have consumed a sufficiently diverse diet if she had consumed at least five of the ten food groups in the 24 h prior to data collection^31,32^. Pregnant adolescents were asked to list every meal they had and drink they had consumed during the preceding 24 h, both inside and outside the home. In addition, the participants were asked to recall any between-meal snacks they may have had. Food groups were scored as "1" for consumption over the reference time and "0" for nonconsumption.

The food security assessment was conducted using the HFIAS Guidelines^32^. The degree of food security in the household was assessed using 27 questions from the Household Food Insecurity Access Scale. The questions were previously authorized for use in developing countries.^33^ Households are labeled food-secured if they experienced fewer than the first two food insecurity indicators. Households that experienced between two and ten, 11 and 17, or more than 17 food insecurity indicators were categorized as mildly, moderately, or severely food insecure, respectively.

The wealth index of the household was determined through the application of principal component analysis (PCA), considering factors such as agricultural land, household possession, animals, water supply, and latrine access. Three categories were created from all the responses for the nondummy variables. The code for the highest rating was 1. Nevertheless, codes of 0 were assigned to two lower numbers. Factor scores in PCA were produced with variables having a commonality value higher than 0.5. The score for each family's first primary component was retained to calculate the wealth score. The quartiles of the wealth score were developed to classify households as the poorest, poor, medium, rich, or richest^34^.

Pregnant teenagers were asked eight questions to gauge their level of autonomy. For every question, code one was provided if the adolescent made the decision on her own or in conjunction with her husband; otherwise, code zero was provided The mean^35^. was used to classify pregnant adolescents’ decision-making abilities.

Maternal knowledge of food intake during pregnancy was assessed using a set of twelve questions. A code of one was given for a correct response, and a code of zero was given for an incorrect response. Pregnant adolescents who scored ≥ 75% on the knowledge questions were considered to have good knowledge; otherwise, they were considered to have a low knowledge score^36^.

### Operational definition

Pregnant adolescent: Pregnant girls within 10–19 years of age^3^.

Nutrition knowledge: is pregnant adolescent knowledge regarding optimal nutrition during pregnancy and it is scored as: Pregnant adolescent who score >  = 75 and > 50% from 12 knowledge questions were labeled as having high and medium knowledge scores, respectively. Otherwise, they are considered to have low knowledge score 27^34^.

### Data quality control

We used a standard-adapted questionnaire and adhered to the steps needed to produce the desired results, and the highest level of data quality possible was ensured. To maintain consistency, the questionnaires were translated into the local languages Amharic and Afan Oromo and then translated back into English. Pretests were also conducted on 23 pregnant adolescents in the Wondo Genet district. A test–retest method was used for reliability analysis of the pregnant adolescent nutrition knowledge variable, a Cronbach coefficient of 0.83 was obtained after pre-test. Based on the gaps found during the pretest, changes were made to the data-gathering methods to improve their reliability. The principal investigator provided a two-day training session for supervisors and data collectors regarding the objective and methodology of the data collection. Supervisors monitored field practices and reviewed the completed surveys daily to ensure data accuracy.

### Data management and statistical analyses

We collected the data using Kobo Collect/Toolbox and exported the data to SPSS version 25 for cleaning and analysis. A bivariate logistic regression model was applied to identify the independent predictors of nutritional knowledge. In the bivariate analysis, variables with p values less than 0.25 were added to the multivariable logistic regression model. The 95% confidence intervals (CIs), crude and adjusted odds ratios (CORs), and AORs were used to calculate the strength of the relationship. According to the multivariate logistic regression analysis, variables with a P value of 0.05 or lower were regarded as independent predictors of nutritional knowledge.

Univariate linear regression analyses were also conducted before the multivariable linear regression analysis. To identify the predictors of MUAC (nutritional status), multivariable linear regression analysis with stepwise variable selection was employed, and variables that were statistically significant (*P* < 0.05) in the univariate analyses were entered into the multivariable linear regression.

The goodness-of-fit of the model was assessed using adjusted R^2^ (R^2^ = 33.8%), and partial regression residual plots showed that all the models had a linear relationship. The normality of the data was assessed using a Q‒Q plot, and there was no need for transformation. Outliers and influential observations were not as influential and were retained in the final model. Collinearity between predictor variables was checked using the variance inflation factor (VIF); the maximum VIF was 1.4, and interaction and confounding variables were checked. All tests were two-sided, and a *p* value < 0.05 was used to indicate statistical significance. We present the results of the linear regression as parameter estimates (β), *P* values, and 95% confidence intervals. The percentage of missing data was low; therefore, the imputation method for missing data management was used.

### Ethics approval and consent to participate

Ethical approval was obtained from the Jimma University IRB/Ethics Committee (reference number JUIH/IRB/194/22). All methods were performed in accordance with the relevant guidelines and regulations; the study was performed in accordance with the Declaration of Helsinki. Before providing informed consent, each study participant provided a detailed description of the study's title, objective, protocol, and duration, as well as its risks and benefits. Each adolescent provided verbal, written, and signed informed consent forms before any measurements or interviews. Participants were made aware of the publication of their anonymous comments. Informed consent was obtained from all participants prior to the commencement of the interviews. The researcher was satisfied with the academic and ethical requirements. Finally, the researcher kept the data in a locked file cabinet in a safe place after the completion of the study. Informed consent was obtained from both the adolescents and their husbands or parents.

## Results

### Sociodemographic characteristics of pregnant adolescents

Of the 459 pregnant adolescents invited to participate in this study, 426 fully participated, for a response rate of 92.8%. The mean (± SD) age of the pregnant adolescents was 18.6 (± 0.74) years. Table 1 presents the sociodemographic characteristics of the participants.

**Table 1:** Sociodemographic characteristics of pregnant adolescents in the West Arsi Zone, central Ethiopia, 2023.

Variable	Frequency	Percent
Age		
Middle adolescent	97	22.7
Late adolescent	329	77.2
Family size		
< 5	321	75.4
> = 5	105	24.6
Marital status		
Married	362	84.9
Unmarried/divorced	64	15.1
Educational status		
No formal education	50	11.7
Can read & write	56	13.1
Primary education	215	50.4
Secondary education	87	20.4
College and above	21	4.9
Occupation		
House wife	135	31.7
Student	163	38.3
Merchant	73	17.1
Daily laborer	16	3.7
Farmer	18	4.2
Government job	21	4.9
Wealth index		
Poorest	88	20.6
Poor	80	18.7
Medium	49	11.5
Rich	129	30.2
Richest	80	18.7

### Nutrition knowledge among pregnant adolescents

Approximately 101 (23.7%) 95% CI (21.4–25.3%) of the study participants had good nutritional knowledge, and 76.3% had poor nutritional knowledge.

### Dietary diversity practice and food security of participants

Twenty one percent of the pregnant adolescents had adequate dietary diversity practice and 78% had inadequate dietary diversity practice during the preceding 24 h of the survey (95% CI (74.3%, 82.8%)). The most frequently eaten foods were pulses (79%) and starchy foods (81.3%) and the least consumed foods were, fruits (3.48%) and meats (2.8%). 319 (74.8%) of the participants are food insecure and 107 (25.2%) are food secured.

### Factors associated with nutritional knowledge among pregnant adolescents

Educational status, occupation of the pregnant adolescent, ANC visits, and nutritional education were significant factors in the bivariable logistic regression analysis, and educational status, ANC visits, and nutritional education remained significantly associated with good nutritional knowledge at the multivariable logistic regression level.

The odds of having good nutritional knowledge were 7.5 times greater among pregnant adolescents whose highest education attained was college education or above than among participants with no formal education [AOR = 7.5, 95% CI = (5.27–9.38)], *P* = 0.002; the odds of having good nutritional knowledge were 8 times greater among pregnant adolescents who had ANC visits than among those who had no ANC visit [AOR = 8, 95% CI = (3.63–13.85)], *P* < 0.001; and the odds of having good nutritional knowledge were 5 times greater among pregnant adolescents who received nutritional education and counseling than among those who did not receive nutritional education [AOR = 5, 95% CI = (3.67–13.53)], *P* < 0.001]. (Table 2).

**Table 2:** Factors associated with nutritional knowledge among pregnant adolescents in the West Arsi Zone, central Ethiopia, 2023 (n = 426).

Variables		Nutrition knowledge		COR	AOR
		Good	Low	(95% CI)	(95%CI)
Educational status	No formal education	9	41	1	1
	Can read and write	14	39	1.63 (1.25–5.75)	1.46 (1.37–4.36)
	Primary education	24	191	0.57 (0.09–0.86)	0.36 (0.17–0.73)
	Secondary education	39	48	3.7 (1.96–8.36)	2.4 (2.17–7.47)
	College and above	15	6	11.3 (4.63–18.74)	7.5 (5.27–9.38)**
Occupation	House wife	12	123	1	
	Student	36	127	2.9 (1.37–8.84)	2.2 (1.64–6.74)
	Merchant	21	52	4.1 (2.28–13.56)	3.3 (1.94–9.37)
	Daily laborer	7	9	7.9 (3.73–16.38)	5.2 (2.38–8.83)
	Farmer	10	8	12.8 (7.28–17.73)	3.6 (2.56–8.64)
	Government job	15	6	25.6 (12.38–31.49)	6.3 (2.68–11.53)
ANC Visit	Yes	74	38	20.7 (13.73–28.72)	8 (3.63–13.85)***
	No	27	287	1	
Nutrition education	Received	82	75	14.4 (6.83–18.47)	5 (3.67–13.53) ***
	Not received	19	250	1	

OR = odds ratio, AOR = Adjusted odd ratio, CI = Confidence interval * * Significant at *P* = 0.002, * ** Significant at *P* =  < 0.001.

The maximum variance inflation factor (VIF) is 1.4

Nutritional education (β = 0.25, *P* = 0.002) and good nutritional knowledge (β = 0.08, *P* < 0.001) were positively associated with MUAC (nutritional status). However, food insecurity (β = − 0.93, P < 0.001) was negatively associated with the MUAC (nutritional status). (Table 3).

**Table 3:** Linear regression model predicting the MUAC nutritional status of pregnant adolescents in the West Arsi Zone, Central Ethiopia, 2023.

Variables	Multivariable linear regression		
	β	95% CI	*P* value
Age (in years)	0.06	[− 0.274, 0.39]	0.759
Husbands education	0.06	[− 0.04, 0.17]	0.474
ANC visit (having visit)	0.28	[0.18.,0.72]	0.083
HH decision making (Females involved)	0.05	]− 0.4,0.52]	0.364
Nutrition education (received)	0.25	[0.06, 0.47]	0.002
Nutrition knowledge (Good)	0.08	[0.02–0.38]	< 0.001
Meal frequency	0.22	[− 0.13, 0.57]	0.073
Food security (food insecure)	− 0.93	[− 2.38, − 0.37]	< 0.001

## Discussion

This study revealed that the magnitude of good nutritional knowledge was 23.7%, and the educational status of the pregnant adolescents, ANC visits, and nutritional knowledge were significantly associated with good nutritional knowledge. Nutrition education and good nutritional knowledge were positively associated with nutritional status, and food insecurity was negatively associated with nutritional status.

The magnitude of good nutritional knowledge in this study (23%) was lower than that in other studies, such as the studies conducted in Ambo, Ethiopia (33%),^37^ Guto Gida, East Wellega, Ethiopia (64.4%)^38^, Accra, Ghana (44.9%),^39^, and Sialkot, Pakistan (47.5%)^40^. This might be because of the differences in study settings (community-based vs. facility-based) and differences in sociodemographic and economic factors. Moreover, these studies included pregnant women and pregnant adolescent participants, whereas the current study included pregnant adolescents who were less mature. The large nutritional knowledge gap reported in this study implies that health care providers, including health extension workers, planners, and community health agents, should work to improve the nutritional knowledge of pregnant adolescents. Nutritional knowledge alone cannot guarantee an individual’s good behavior, but it is a predisposing factor that can significantly shape their attitudes, which can be reflected in a person’s actions.

The odds of having good nutritional knowledge were 7.5 times greater among pregnant adolescents whose highest education attained was college education or above than among participants with no formal education; this finding is consistent with several studies, such as the studies conducted in Ambo, Ethiopia^37^, East Wellega, Ethiopia,^38^ Addis Ababa, Ethiopia,^41^, and Klang Valley, Malaysia^42^; This could be because pregnant adolescents with higher education levels can more effectively use textual resources for information, such as books, newspapers, flyers, Internet articles and other educational materials. Less educated mothers deserve more attention, and they should receive intensive nutrition education in simple language and ways they can understand.

The odds of having good nutritional knowledge were eight times greater among pregnant adolescents who had ANC visits; this finding is in agreement with the findings of Addis Ababa, Ethiopia^41^, the Harari region in Eastern Ethiopia^42^, and Beirut and Lebanon^43^. The ANC programme offers resources to support preventative healthcare behaviors, enhance health literacy, and enhance nutrition. During their ANC appointments, pregnant women have plenty of time to talk about eating healthily. There is low pregnancy attendance in ANC services in Ethiopia^44^, and this need needs to be improved to equip pregnant women with the necessary nutritional education and other health services.

The odds of having good nutritional knowledge were five times greater among pregnant adolescents who received nutrition education and counseling than among participants who did not receive nutrition education, which is consistent with the findings of a study conducted in Ambo, Ethiopia^37^. Nutritional education is essential for improving food and nutritional literacy in pregnant women. The nutritional knowledge of pregnant individuals can be directly improved by providing nutritional and health information; pregnant adolescents need to be given sufficient nutritional education at both the facility and community levels to improve their dietary practices, which ultimately results in good pregnancy outcomes.

Good nutritional knowledge was positively associated with the nutritional status of pregnant adolescents, which is in agreement with the findings of previous studies in the Silte zone, southern Ethiopia^45^, and Klang Valley, Malaysia^46^. People’s literacy about the consumption of food is very significant and determines their food choices and habits. Nutritional knowledge is an important determinant of good dietary practices^47,48^, and good dietary practices can lead to better nutritional status^49,50^.

Receiving nutrition education was positively associated with the nutritional status of pregnant adolescents, which is similar to the findings of a study conducted in Dessie Town, northeastern Ethiopia^51^. Nutrition education can enhance pregnant adolescents’ understanding of optimal nutrition, leading to an improvement in their nutritional status. Their increased understanding will enable them to begin healthy eating habits, which in turn will improve their nutritional condition.

Being food insecure was negatively associated with the nutritional status (measured by MUAC) of pregnant adolescents, which is consistent with the findings of studies conducted in Ambo, Ethiopia^37^, rural Ethiopia^52^, and a systematic review conducted globally^53^. Food insecurity or eating food that does not contain all the essential nutrients for good nutritional status is an immediate cause of undernutrition. There is a commitment to advancing maternal and child nutrition across states through the 2030 Sustainable Development Goals^54^; preventing and managing food insecurity is very important for achieving this global goal.

In this community-based study, pregnant adolescents were visited at their homes to provide primary data. Recall bias could exist because of the respondents' potential to forget certain facts. However, to reduce recall bias, the data collectors received training on how to elicit information from pregnant adolescents. However, further studies are needed to investigate the magnitude of undernutrition based on validated specific cut-off point for pregnant adolescent.

## Conclusion

The nutritional knowledge of pregnant adolescents was suboptimal; educational status, ANC visits, and receiving nutrition education were associated with good nutritional knowledge, whereas food insecurity, low nutritional knowledge, and not receiving nutrition education were predictors of poor nutritional status. Nutritional education and counseling interventions, increasing the utilization of ANC, and interventions for improving food security and randomized control trail using nutrition behavioral change communications are strongly recommended.

## Data Availability

The datasets used and/or analyzed during the current study are available from the corresponding author.
